# Depression in polycystic ovary syndrome: Focusing on pathogenesis and treatment

**DOI:** 10.3389/fpsyt.2022.1001484

**Published:** 2022-08-31

**Authors:** Liwei Xing, Jinlong Xu, Yuanyuan Wei, Yang Chen, Haina Zhuang, Wei Tang, Shun Yu, Junbao Zhang, Guochen Yin, Ruirui Wang, Rong Zhao, Dongdong Qin

**Affiliations:** ^1^The First School of Clinical Medicine, Yunnan University of Chinese Medicine, Kunming, China; ^2^Department of TCM, Yunnan Maternal and Child Health Care Hospital, Kunming, China; ^3^School of Basic Medical Sciences, Yunnan University of Chinese Medicine, Kunming, China; ^4^Department of TCM, Hainan Women and Children's Medical Center (Women and Children's Health Care Center of Hainan Province, Hainan Children's Hospital, Children's Hospital of Fudan University at Hainan, Hainan Obstetrics and Gynecology Hospital), Haikou, China; ^5^Department of Journal Editorial, Yunnan University of Chinese Medicine, Kunming, China; ^6^Department of Acupuncture and Moxibustion, Kunming Municipal Hospital of Traditional Chinese Medicine, Kunming, China; ^7^The Second School of Clinical Medicine, Yunnan University of Chinese Medicine, Kunming, China; ^8^School of Chinese Materia Medica, Yunnan University of Chinese Medicine, Kunming, China

**Keywords:** polycystic ovary syndrome, depression, pathogenesis, mechanism, treatments

## Abstract

Polycystic ovary syndrome (PCOS) is one of the most prevalent gynecological endocrine conditions affecting reproductive women. It can feature a variety of symptoms, such as obesity, insulin resistance, skin conditions, and infertility. Women with PCOS are susceptible to illnesses including mood disorders, diabetes, hypertension, and dyslipidemia. Among them, depression is the most common in PCOS and has a detrimental effect on quality of life. Depression may occasionally develop due to the pathological traits of PCOS, but its exact pathogenesis in PCOS have eluded researchers to date. Therefore, there is an urgent need to explore the pathogenesis and treatments of depression in PCOS. The present review discusses the epidemiology of depression in PCOS, potential pathogenic mechanisms underlying PCOS and depression, as well as some potential factors causing depression in PCOS, including obesity, insulin resistance, hyperandrogenism, inflammation, and infertility. Meanwhile, some common treatment strategies for depression in PCOS, such as lifestyle intervention, acupuncture, oral contraceptive pills, psychological intervention, and insulin-sensitizer, are also reviewed. To fully understand the pathogenesis and treatment of depression in PCOS, a need remains for future large-scale multi-center randomized controlled trials and in-depth mechanism studies.

## Introduction

Polycystic ovary syndrome (PCOS) is a common disease in women and is characterized by hyperandrogenemia, chronic anovulation, and polycystic ovary morphology (1). Endocrine and energy metabolism disorders lead to a variety of clinical symptoms of PCOS, including anovulation and amenorrhea (75–80%), infertility (75%), excessive hair growth (70%), and obesity (50%) (2). The prevalence of PCOS is rising and can range from 5 to 20% depending on the demographic studied and the diagnostic criteria applied (3). Patients with PCOS experience significant social and financial pressure, which increases the prevalence of mental illnesses such as anxiety and sadness. According to a recent study, anxiety and sadness are prevalent in women with PCOS with rates of 38.6 and 25.7%, respectively (4). The most prevalent cause of disability globally is depression, which is three to eight times higher in women with PCOS than in control groups (5, 6). Global standards indicate that all PCOS patients should be screened for depression at diagnosis, reflecting growing concern about this aspect of the condition (7). Obesity, insulin resistance, hyperandrogenism, inflammation, and infertility—all pathogenic aspects of PCOS—have been linked in studies to the emergence of depression. This review discusses the epidemiology of depression in PCOS, potential pathogenic mechanisms underlying the association between depression and PCOS, and some common treatment strategies for depression in this condition.

## Depression in PCOS

### Epidemiology

Using the PHQ-9 (patient health questionnaire-9), 64.1 percent of women with PCOS have been found to have depressive disorders, a significantly higher proportion than in a non-PCOS group (*P* < 0.01). In PCOS patients, the odds ratio for depressive disorders was 5.7, and univariate analysis showed significantly higher body mass index (BMI) in women with than without PCOS. Age, marital status, educational attainment, and place of employment did not significantly influence the onset of depression (8). Other research has shown that depression is more likely to occur in black than white women with PCOS (*P* < 0.001) (9). In comparison to non-PCOS counterparts, the prevalence of depression in PCOS was considerably higher in the overweight and obese categories (PCOS 33.2% vs. non-PCOS 16.2%; *P* < 0.001) (10). Women with PCOS also have a higher incidence of depression-related hospitalizations than those without PCOS (11). The prevalence of PCOS-related depression in various geographic areas is displayed in Table 1.

**Table 1:** Summary of studies indicating prevalence by geographic region and examining the impact of PCOS-related treatments on depression in randomized controlled trials.

**Type**	**Country and case inclusion period**	**Prevalence (%)**	**Groups (number of subjects)**	**Treatment**	**Treatment length**	**Assessment scales**	**Results**	**References**
Summary of studies indicating prevalence of depression in PCOS by geographic region	United States, 2005–2008	64.1%	PCOS with depression (*n* = 75), PCOS without depression (*n* = 42)	/	35 months	PHQ-9	The prevalence of depressive disorders among women with PCOS was 64.1%	(8)
	United States, 1985–1986	36%	No PCOS (*n* = 1,044), PCOS (*n* =83)	/	12 months	CESD	CES-D scores were higher among women with PCOS, and black women experienced higher depression burden than white women	(9)
	Australia, 1973–1978	27.3%	PCOS (*n* = 478), non-PCOS (*n* = 8,134)	/	60 months	CESD-10	Women with PCOS, reported higher prevalence of depression than women without PCOS (27.3 vs. 18.8%)	(10)
	Korean, 2007–2010	15.35%	PCOS (*n* = 26,251), Non PCOS (*n* = 131,480)	/	36 months	/	The risk of developing depression in women with PCOS was higher compared to women without PCOS	(11)
	Syria and Jordan, 2017	83% in Syria and 65% in Jordan	Syria (active, *n* = 30 vs. control, *n* = 30), Jordan (active, *n* = 30 vs. control, *n* = 28)	/	5 months	Beck depression inventory	Syria and Jordan highlighted a high prevalence of depression (Syria = 83% vs. Jordan = 65%)	(12)
Randomize-d controlled trials assessing the effect of PCOS-related treatments on depression in women with PCOS	Netherlands, 2010–2016	/	CAU (*n* = 60). CBTLS (*n* = 63) CBTLS+SMS (*n* = 60)	Cognitive behavioral lifestyle sessions combined with a healthy diet and physical therapy	12 months	BDI-II, RSES, FNAES	A three-component lifestyle intervention based on CBT could improve depression in women with PCOS	(13)
	United States, 2013–2015	/	CBT+LS (*n* = 20), LS (*n* = 13)	Cognitive-behavioral therapy (CBT) and lifestyle modification (LS)	16 weeks	CESD, STAI	CBT+LS significantly improved depressive symptoms in women with PCOS compared with LS alone	(14)
	China, 2018–2019	/	Intervention group (*n* = 61), control group (*n* = 61)	Transtheoretical model-based mobile health application intervention program	12 months	SAS, SDS	TTM-based mobile health application program can decrease depression in patients with PCOS	(15)
	Australia, not mentioned	/	HPLC: (*n* = 14); LPHC: (*n* = 14)	High-protein, low-carbohydrate diet (HPLC)	16 weeks	HADS and the Rosenberg Self Esteem Scale	The HPLC diet was associated with significant reduction in depression	(16)
	Brazil, 2014–2016	/	CAT (*n* = 23), IAT (*n* = 22), CG (*n* = 24)	Continuous and intermittent aerobic physical training	16 weeks	HADS	Both CAT and IAT groups had significant reductions in depression scores	(17)
	China, 2016–2019	/	A (*n* = 20). LS (*n* = 20).	Acupuncture	4 months	SAS, SDS	Acupuncture can effectively relieve depression in patients with PCOS, and the mechanism may be related to the regulation of serum β-endorphin and androgen	(18)
	Swedish, 2005–2008	/	Acupuncture (*n* = 28); exercise (*n* = 29); control (*n* = 15)	Acupuncture	16 weeks	MADRS-S, BSA-S	Acupuncture can lead to a modest improvement in depression scores in women with PCOS	(19)
	China, 2012–2016	/	Acupuncture group (*n* = 27), sham acupuncture group (*n* = 27)	Acupuncture	16 weeks	Zung-SAS and Zung-SDS	Acupuncture can influence serum levels of NE and 5-HT, improving symptoms of depression in PCOS patients	(20)
	United States, 2008–2014	/	OCP group (*n* = 45), LS group (*n* = 44), combined group (*n* = 43)	Oral contraceptive pills (OCPs; ethinyl estradiol/norethindrone acetate)	16 weeks	Positive screens on the Prime-MD	OCPs result in significant improvements in depressive symptoms	(21)
	Athens, 2012–2013	/	Intervention group (*n* = 23), control group (*n* = 15)	Mindfulness stress management program	8 weeks	DASS 21	Mindfulness techniques ameliorate stress, anxiety, depression and the quality of life in women with PCOS	(22)
	Danish, 2014–2016	/	MI+ SA (*n* = 19), SA (*n* = 18)	Motivational interviewing	6 weeks	WHO-5 and MDI	Motivational interviewing can significantly improve depression scores	(23)
	Germany, 2011–2012	/	Pioglitazone (*n* = 20), metformin (*n* = 20)	Pioglitazone	6 weeks	HDR-17	Pioglitazone improves depression with mechanisms largely unrelated to its insulin-sensitizing action	(24)
	China, 2016–2018	/	PM (*n* = 28), M (*n* = 26), placebo (*n* = 21)	Pioglitazone metformin complex preparation (PM)	12 weeks	SCL-90-R	Pioglitazone metformin alleviates depression *via* inhibiting NLRP3 inflammasome	(25)

### Pathogenesis of PCOS

Hypothalamic-pituitary-gonad dysfunction is thought to be the primary cause of PCOS. Gonadotropin-releasing hormone (GnRH) is pulse-released in the hypothalamus under typical circumstances. Increased frequency of release encourages luteinizing hormone (LH) to be released from the anterior pituitary gland, while reduced frequency encourages the production of follicle stimulating hormone (FSH) (26). The amounts of GnRH, LH, and FSH secreted by the pituitary and hypothalamus are controlled by the levels of estrogen, progesterone, androgen, and other steroid hormones generated by the ovary. In PCOS patients, dysfunction in the GnRH neuronal network in the brain resulted in decreased responsiveness to gonadal steroid hormone negative feedback, destroying the afore-mentioned feedback loop and causing rises in GnRH pulse frequency, increasing LH pulse frequency, decreasing FSH pulse frequency, and abnormally increasing the LH/FSH ratio (27). Ovarian granulosa cells' reaction to reduced FSH release frequency leads to a distortion in the selection of dominant follicles, with follicle stagnation, a polycystic state detected on ultrasound examination, and infertility (28). However, elevated LH causes theca cells in the ovary to produce excessive androgen, which causes hyperandrogenemia (29). In addition, peripheral estrogen synthesis occurs as a result of high serum androgen levels in PCOS (30). The pro-inflammatory nature of PCOS is caused by excess estrogen, which increases the synthesis of a variety of inflammatory cytokines (31). Inflammatory markers IL-6 and tumor necrosis factor alpha (TNF-α) are linked to insulin resistance, another metabolic abnormality which may be induced in PCOS (32).

### Pathogenesis of depression

The pathogenesis of depression is associated with alterations in the hypothalamic-pituitary-adrenal (HPA) axis (hypothalamus, pituitary, adrenal and downstream target organs) and a decrease in monoamine neurotransmitter level (33). Corticotropin releasing hormone, adreno-cortico-tropic-hormone and cortisol secretion increase when the body is under psychological stress (such as competition for a job) (34). Excessive cortisol inhibits HPA axis activity, maintaining body hormone homeostasis. However, if stress persists, cortisol will remain high, leading to desensitization of cortisol receptors and further stimulation of the HPA axis, which eventually destroy the negative feedback regulation between cortisol and its receptors, causing continuous hyperactivity of the HPA axis, and forming a vicious cycle leading to depression (35). Decreased secretion of serotonin, acetylcholine and other neurotransmitters also negatively affects function of the HPA axis and similarly leads to depression (33).

### Pathogenesis of depression in PCOS

**Figure 1:**
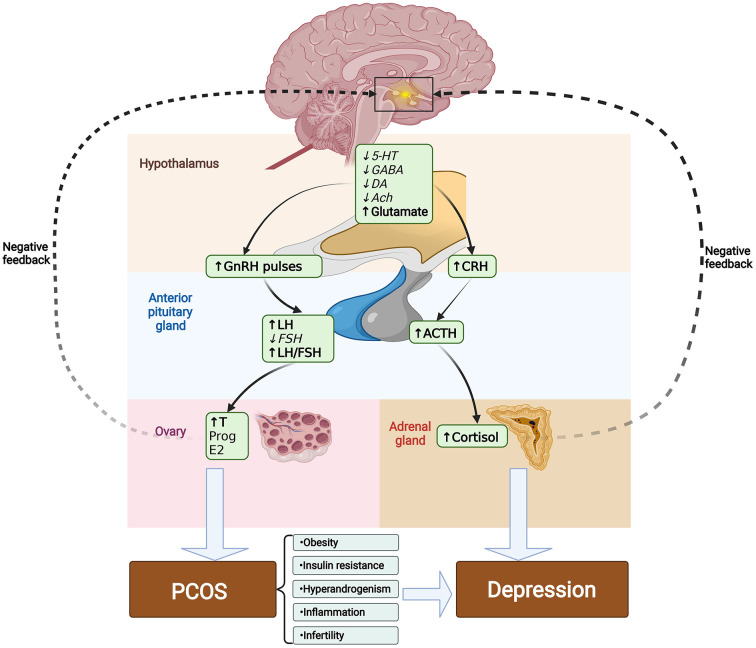
Changes in neurotransmitters may be involved in the pathogenesis of PCOS-induced depression. In PCOS, GnRH and LH inhibitory neurotransmitters such as 5-HT, DA, GABA and Ach are decreased. While the major stimulants of GnRH and LH such as glutamate are increased. Elevated frequency of release in GnRH encourages LH to be released from the anterior pituitary gland, while reduced frequency encourages the production of FSH, which abnormally increases the LH/FSH ratio. Elevated LH causes theca cells in the ovary to produce excessive androgen and eventually exacerbates the progression of PCOS. Decreased secretion of serotonin, acetylcholine and other neurotransmitters also negatively affects function of the HPA axis, which increases the levels of CRH, ACTH, and cortisol, causing continuous hyperactivity of the HPA axis, and leading to depression. Meanwhile, the pathological traits of PCOS, including obesity, insulin resistance, hyperandrogenism, inflammation, and infertility can exacerbate the onset of depression. Italic font indicates lower levels compared to normal, whereas bold font indicates higher levels. 5-HT, serotonin; DA, dopamine; GABA, gamma-aminobutyric acid; Ach, acetylcholine; GnRH, gonadotropin-releasing hormone; LH, luteinizing hormone; FSH, follicle stimulating hormone; T, Testosterone; E2, estradiol; CRH, corticotropin releasing hormone; ACTH, adreno-cortico-tropic-hormone.

Inhibitory neurotransmitters such as serotonin (5-HT), dopamine (DA), gamma-aminobutyric acid (GABA), and acetylcholine (Ach) are diminished in PCOS. In contrast, glutamate levels, which are the primary stimulants of GnRH and LH, are raised in PCOS-related disorders, and these neurotransmitter alterations could play a part in the pathophysiology of depression in PCOS (36). The specific mechanisms driving the higher prevalence of depressive symptoms in PCOS-positive women are not yet established, however there are many possible contributing factors: obesity, insulin resistance, hyperandrogenism, inflammation, and infertility (see Figure 1).

### Obesity

Up to 80% of women with PCOS have morbid obesity (37). An increased depression score and a higher likelihood of depression were significantly correlated in obese women with PCOS (38); The HPA axis is dysregulated in obesity, with extensive over-production of cortisol (39). In PCOS, abnormally high levels of systemic cortisol lead to the overexpression of mineralocorticoid and glucocorticoid receptors, which in turn reduces negative feedback and ultimately results in a state of sustained hypercortisolism (40). Excessive secretion of cortisol and impaired glucocorticoid-mediated feedback mechanism result in the development of depression in PCOS (41). Additionally, by activating the highly expressed glucocorticoid receptors in the visceral and intra-abdominal areas, cortisol stimulates the deposition of central fat (42) and promotes lipid accumulation, which leads to visceral and central obesity and, ultimately, metabolic disorders (43) in a vicious cycle. Additionally, the obese patient's perception that they are physically unattractive may exacerbate depressive symptoms and their perception of their own femininity (44). In conclusion, obesity, as a result of abnormal metabolism of PCOS, is closely related to the dysfunction of HPA axis, leading to the development of depression.

### Insulin resistance

About 75% of women with PCOS meet the World Health Organization (WHO) criteria for insulin resistance (45). According to one study, insulin resistance may act as a physiological mediator and has an independent relationship with depression in PCOS, with Homeostatic Model Assessment of Insulin Resistance score linked to a 2.3-fold higher risk of depression (46). One proposed molecular reason for the emergence of depression through insulin resistance is elevated cortisol, accompanied by elevated sympathetic nerve activity and decreased levels of 5-HT in the central nervous system (47). 5-HTs are potent neurotransmitters with a broad range of effects (48). Depression can result from diminished central 5-HT function (49). Decreased level of 5-HT in patients with depression can antagonize the direct inhibition of insulin secretion, leading to insulin dysfunction and insulin resistance (50), thus forming a vicious cycle between insulin resistance and depression.

### Hyperandrogenism

A large majority of PCOS-affected women has clinical and/or biochemical hyperandrogenism (47). Clinically, hyperandrogenism can manifest as hirsutism, acne, and alopecia (51). When compared to women without depression in PCOS, those with depression in PCOS had higher levels of free testosterone, according to a meta-regression analysis (52). Hyperandrogenemia associated with obesity, hirsutism, acne and hair loss may alter self-image, which can lead to depression (53). It is reported that the serum concentrations of 5-HT, 5-hydroxyindoleacetic acid (5-HIAA), and the DA metabolite homovanillic acid were significantly lower while the testosterone and the depressive subscales were significantly higher in women with PCOS compared to those without PCOS, suggesting a relationship among hyperandrogenism, depression and altered neurotransmitter contents in PCOS (54). Animal studies have also demonstrated that dehydroepiandrosterone caused depression-like behavior in mice with PCOS, potentially *via* down-regulation of brain monoamines and associated metabolites, indicating a role of hyperandrogenism in the mental health conditions found in PCOS (55). Above all, the hyperandrogenism associated with PCOS that results in depression may be based on the monoamine hypothesis of depression.

### Inflammation

Previous research has shown that PCOS is an inflammatory condition (56). Various studies have shown elevated serum levels of TNF-α in PCOS (32, 52). In addition, high estrogen release during PCOS increases interleukin 4 (IL-4), IL-1, IL-6, and interferon γ (IFN-γ) production (31) and these increases are associated with depression-like symptoms in human and animal studies (57). When this cytokine signal enters the brain, depression may result, having a noticeable impact on brain monoamines like 5-HT and DA. Tryptophan, the major precursor for 5-HT production, is depleted when cytokines activate the enzyme indoleamine 2,3-dioxygenase, which metabolizes the conversion of tryptophan into kynurenine. This results in lower numbers of 5-HT receptors in the brain (56). Cytokines may potentially affect the availability of 5-HT by interfering with synaptic reuptake through use of presynaptic transporters such as the high-affinity 5-HT transporter (58). For instance, it has been observed that IL-1 and TNF activate p38 mitogen-activated protein kinase, which results in the phosphorylation and increased expression of 5-HT reuptake pumps, increasing the intake of 5-HT and, consequently, the manifestation of depressive symptoms (59). Depression also increases inflammation. Stress promotes the expression of cytokines in the brain and peripheral areas of the body, activates microglia, and causes an inflammatory response through the sympathetic nervous system (60). Furthermore, major depressive disorder intensifies the body's inflammatory reaction to stress. Proinflammatory cytokines (such IL-1 and IL-6) are linked to both acute and chronic stress, and affect the severity and speed of development of depression (61). To sum up, excessive inflammation caused by PCOS is an important inducer of depression.

### Infertility

Up to 72% of patients with PCOS experienced infertility (2), of which the clinical pregnancy rate using *in vitro* fertilization and embryo transfer (IVF-ET) technology is only 29% (62). The treatment of infertility may impact emotions through the interaction of estrogen and/or progesterone and serotonin and worsen the incidence of depression in women with PCOS due to the high social, family, and economic pressures they confront (63). According to one study, 40.8% of infertile women suffer from depression, which is associated with the duration of infertility and is most prevalent 4–6 years after diagnosis (26). Another study reported that, the fertility problem inventory subscale scores and the overall stress scale score in the infertility patients were negatively correlated with clinical pregnancy outcome, indicating that the higher the pressure, the lower the pregnancy success rate of IVF-ET (64). Two common immunoendocrine mechanisms that underpin infertility may act as mediators between psychopathology/stress and poor reproduction. Infertility has been linked to immunological imbalance, which can lead to parasecretion of hormones, cytokines, and neuropeptides. These offer a typical molecular pathogenesis for depression in PCOS (65).

### Treatment

No specific drug treatment has been identified for PCOS depression to date, and most studies in this field have assessed the effectiveness of PCOS-specific treatment regimens. Randomized controlled trials on effectiveness are summarized in Table 1.

### Lifestyle intervention

For patients with PCOS, healthy lifestyle is the primary means of treatment, such as the combined application of diet and exercise. One study reported that diet, exercise, and cognitive behavioral therapy (CBT) combined with a lifestyle intervention program significantly improved depression and self-esteem in patients with PCOS, and weight loss was positively associated with self-esteem (13). Another randomized controlled study including 33 overweight/obese women with PCOS and depressive symptoms found that weekly CBT and lifestyle change for 8 weeks significantly reduced weight and enhanced quality of life compared to lifestyle change alone. Both groups experienced a decrease in heart rate at 8 weeks, which could be interpreted as an improvement in sympathetic responses to stress or habituation triggered by repeated measurements. This finding raises the possibility of a connection between CBT, weight loss, and regulation of the stress response (14). Dietary modifications have a substantial impact on PCOS, and studies have shown that adopting a high-protein, low-carbohydrate (HPLC) diet is linked to a considerable decline in depressive symptoms and an increase in self-esteem. It is likely that HPLC diets are associated with better compliance and, therefore, more effectiveness in the long-term treatment of obesity due to a greater sense of wellbeing (16). Exercise can regulate PCOS patients' body weight, endocrine function, depression, and other factors. Anxiety and sadness ratings dropped significantly in women with PCOS undertaking continuous and intermittent aerobic physical exercise, according to a randomized controlled trial assessing the effects on sexual function and mood in these patients. This finding may be related to the decline in testosterone level (17).

### Acupuncture

Acupuncture therapy is widely used in the treatment of depression (66), and has good efficacy in alleviating PCOS symptoms (67). One study reported reduced BMI, Ferriman-Gallway score, self-rating anxiety scale (SAS) and self-rating depression scale (SDS) scores, while PCOS health-related quality of life questionnaire scores, serum sex hormone binding globulin (SHBG) levels and β -endorphin levels were elevated after acupuncture intervention in PCOS patients. These results indicated that acupuncture therapy can effectively relieve depression in PCOS patients, and the mechanism may be related to the regulation of serum β-endorphin and androgen levels (18). Physiology, energy/vitality, general health impression, and mental component scores in the medical outcomes study short-form 36 (SF-36) domain of PCOS patients in the acupuncture group improved after intervention and during follow-up, according to a secondary analysis from a randomized controlled trial. And the effects of acupuncture persisted for at least 4 months after treatment ended (19). Another secondary investigation to assess how electroacupuncture affected anxiety and sadness in unmarried women with PCOS found that acupuncture could alter serum norepinephrine (NE) and 5-HT levels and thereby lessen PCOS depression symptoms (20).

### Oral contraceptive pills

For women with PCOS, hormonal contraceptives are the primary line of treatment to control menstruation. OCP use was linked to a significant improvement in the psychological component of health-related quality of life in a survey of more than 1,000 women in the general population (68). Studies have shown that low-dose hormonal contraceptive therapy combined with lifestyle changes can improve the psychosocial functioning of PCOS in overweight/obese women and significantly reduce depressive symptoms through a mechanism associated with reduced androgen levels, in addition to well-established benefits like improved menstrual cycle and reduced hirsutism (21). However, few studies on the effects or mechanisms of oral contraceptives on depression in PCOS, and larger and longer-term studies are required to investigate these.

### Psychological intervention

Official guidelines stated that medical staff should evaluate psychological status and provide constructive criticism and adjustments to remove the psychological barriers for patients with PCOS. When necessary, they should also consult with support or guidance groups for reasonable psychological support and intervention in patients with obesity (69). After practicing mindfulness-based stress reduction, blood pressure, blood glucose, emotional distress, and quality of life all improve in women with PCOS (70). Motivational interviewing as a tool for addiction therapy, is now being used to treat psychological disorders and obesity (71, 72). One study revealed that the World Health Organization 5 Wellbeing Index (*P* = 0.028) and Major Depression Inventory (*P* = 0.008) scores were considerably raised when motivational interviewing was supplemented with conventional guidance (23). Large trials are required to augment the evidence on psychological intervention for depression in women with PCOS due to the limited sample sizes and trial periods in the existing published studies.

### Insulin-sensitizer

Insulin sensitizers are used to treat insulin resistance associated with PCOS. Pioglitazone is a thiazolidinedione (TZD), which is an insulin sensitizer. TZDs have been found in numerous animal and human studies to be effective in treating neurological and psychiatric conditions like depression and to have strong antidepressant properties (73, 74). Pioglitazone outperformed metformin in terms of reduced depression [38.3 vs. 8.3% reduction from baseline scores, *F*_(1,37)_ = 73.513, *P* < 0.001] (24). According to one study, pioglitazone metformin can successfully suppress the activation of NLRP3 inflammasome, lessen the release of pro-inflammatory cytokines, and enhance many indicators, including total testosterone, leading to a reduction in psychological distress in PCOS (25).

## Summary and outlook

The mechanism underlying the rising prevalence of depression in PCOS may be related to the pathological traits of PCOS, including obesity, insulin resistance, hyperandrogenism, inflammation, and infertility. However, rather than focusing on the biological mechanisms of depression in PCOS, contemporary research has largely concentrated on the similarities and correlations between the pathological features of PCOS and depression. Meanwhile, there are few randomized controlled trials that deeply study the effective treatment strategy and explore the mechanism. According to previous studies, PCOS-induced depression can be effectively treated with acupuncture, oral contraceptives, psychological therapy, and insulin sensitization agents. Nevertheless, effective data supporting the use of these medications to specifically target depression in PCOS are yet lacking due to varying methodologies and a dearth of randomized controlled trials with a large sample size. To fully understand the pathogenesis and treatment of depression in PCOS, a need remains for future large-scale multi-center randomized controlled trials and in-depth mechanism studies.

## Author contributions

All authors listed have made a substantial, direct, and intellectual contribution to the work and approved it for publication.

## Funding

This study was supported by the National Natural Science Foundation of China (31960178, 82160923, and 82060895), Applied Basic Research Programs of Science and Technology Commission Foundation of Yunnan Province (2019FA007), Key Laboratory of Traditional Chinese Medicine for Prevention and Treatment of Neuropsychiatric Diseases, Yunnan Provincial Department of Education, Scientific Research Projects for High-level Talents of Yunnan University of Chinese Medicine (2019YZG01), Young Top-Notch Talent in 10,000 Talent Program of Yunnan Province (YNWR-QNBJ-2019-235), the Yunnan University of Chinese Medicine Joint Special Project of Applied Basic Research (2019FF002-004), and Yunnan Science and Technology Department Project (202103AC100005).

## Conflict of interest

The authors declare that the research was conducted in the absence of any commercial or financial relationships that could be construed as a potential conflict of interest.

## Publisher's note

All claims expressed in this article are solely those of the authors and do not necessarily represent those of their affiliated organizations, or those of the publisher, the editors and the reviewers. Any product that may be evaluated in this article, or claim that may be made by its manufacturer, is not guaranteed or endorsed by the publisher.
